# Knowledge and Utilisation of Intermittent Preventive Treatment of Malaria among Pregnant Women in Muramvya Health District, Burundi, 2018

**DOI:** 10.24248/eahrj.v4i1.625

**Published:** 2020-06-26

**Authors:** Edouard Nkunzimana, Mu'awiyyah Sufiyan Babale

**Affiliations:** a Ministry of Public Health and Fight against AIDS, Department of Pharmacy, Medicine and Laboratories, Bujumbura, Burundi; b Ahmdadu Bello University, Faculty of Clinical Sciences, Department of Community Medicine, Zaria, Nigeria

## Abstract

**Background:**

Intermittent Preventive Treatment in pregnancy (IPT_p_) of malaria is a key component of malaria control strategy in Burundi. *Sulfadoxine-pyrimethamine* (*SP*) is the drug of choice. Despite the evidence of the effectiveness of IPT_p_ strategy using *SP* in reducing the adverse effects of malaria during pregnancy, the uptake and coverage in Burundi is low. This study was carried out to assess the knowledge and utilisation of IPT_p_ among pregnant women of Muramvya Health District and determine factors that influence the uptake.

**Methods:**

This was a community based cross sectional study conducted from 16^th^ to 28^th^ September 2018. A total of 370 pregnant women were recruited from selected settlements of MURAMVYA Health District. A structured questionnaire was administered to elicit information on socio-demographic characteristics, knowledge, and utilisation of IPT_p_. Epi–Info 7.2.2.6 and Microsoft Excel 2016 software was used to perform univariate, bivariate and multivariate analyses.

**Results:**

Among the 370 pregnant women, 310 (83.8%) had taken IPT_p_–SP at least once in the index pregnancy. However, only 76 (24.5%) had completed the minimum required three doses. Having formal education (aOR=2.5, 95% CI [1.2–5.2], P= .016), parity (aOR=2.1, 95% CI [1.1–4.2], P = .033), and living at less than 5 km from the health facility (aOR=4.1, 95% CI [1.7–9.6], P =0.001) were found to be independent determinants of utilisation (at least one) of IPT_p_–SP. Also, having formal education (aOR=5.0, 95% CI [2.1–24.3], P<.001), and gestational age at first ANC visit (aOR=3.3, 95% CI [1.4–7.7], P=.005) were found to be independent determinants of taking optimal dose (three+) of IPT_p_–SP in Muramvya Health District.

**Conclusion:**

The findings of this study show the low rate of pregnant women receiving the optimal dose of IPT_p_–SP. The study established that the major factors for IPT_p_–SP utilisation are; educational level, distance from home to the health facility, parity and the gestational age at the first ANC visit. It is therefore recommended that healthcare providers in Muramvya district should intensify sensitization and awareness campaign on the importance of girl child education and early ANC attendance in order to increase uptake and utilization of IPT_p_-SP for improved health outcomes.

## BACKGROUND

Malaria is an infectious disease caused by *protozoan* parasites from the members of *Plasmodium* genius that can be transmitted by the bite of infected female Anopheles mosquito or by a contaminated needle or transfusion.^1^ 4 major parasite species are known to cause disease in humans, namely *Plasmodium falciparum, Plasmodium vivax, Plasmodium ovale* and *Plasmodium malariae. Plasmodium knowlesi,* originally known to cause simian malaria, is now recognized as the 5th human malaria parasite.^2^ Malaria due to *Plasmodium falciparum* is the deadliest of all types.^3^ Globally, an estimated 3.4 billion people in 92 countries are at risk of being infected with malaria and 1.1 billion are at high risk (>1 in 1000 chance of getting malaria in a year). According to the World Malaria Report 2018, there were 219 million cases of malaria globally in 2017 (uncertainty range between 203 to 262 million) and 435,000 malaria deaths, representing a decrease in malaria cases and deaths rates of 18% and 28% since 2010, respectively. The burden was heaviest in the WHO African Region, where an estimated 93% of all malaria deaths occurred, and in children aged under 5 years, who accounted for 61% of all deaths.^4^ In Burundi, according to data from the National Health Information System (NHIS), malaria is the main cause of morbidity and mortality recorded in health facilities in the general population. It is responsible for 67.1% of the reasons for consultations in the country, and 60% of deaths in hospitals are due to malaria. The incidence rate went from 32.7% in 2010 to 81.5% in 2017. The prevalence rate is estimated at 50.5% in children under 5 years.^5^ Pregnant women compared to non–pregnant women are at increased risk of malaria infection and the severity of clinical manifestation experienced by these women and their foetus depend on the level of pre–pregnancy immunity. *Plasmodium falciparum* malaria during pregnancy is a well–known cause of maternal and foetal morbidity and mortality and it is clearly an important contributor to both maternal anaemia and low birth weights.^7^

Subsequently, pregnant women suspected of having malaria should be assessed and treated in accordance with national protocols. The consequences of malaria during pregnancy vary with transmission intensity. When the transmission is high, maternal anaemia is common, and on the infant, low birth weight due to foetal growth restriction and/or premature delivery is frequent. In low transmission areas, when non–immune pregnant women become infected, malaria infection may become severe and life–threatening, requiring emergency treatment. Maternal complications include acute lung injury, severe Hypoglycaemia and coma while pregnancy loss due to miscarriage or stillbirth is also frequent. Most studies conducted in sub–Saharan Africa showed that approximately 25 million pregnant women are at risk *of P. falciparum* infection every year. One in four women has evidence of placental infection at the time of delivery.^9^*Plasmodium falciparum* infections during pregnancy in Africa rarely result in fever and therefore remain undetected and untreated.^10^

Malaria infection during pregnancy causes an estimated 900,000 low birth weight deliveries worldwide and may contribute to 100,000 infant deaths annually.^11^ In low–risk zones, episodes of severe malaria are significantly associated with stillbirths, spontaneous abortion, premature delivery, and maternal death. Although pregnant women in malaria–endemic areas have a higher rate of *parasitemia* compared to non– pregnant women, in some cases, infection is largely asymptomatic because some degree of pre–existing immunity is retained during pregnancy.^12^ However, malaria immune women are still susceptible to placental malaria because malaria parasites may become sequestered in the placenta and peripheral blood smears may fail to show evidence of infection.^13^ Both situations are conducive for low birth weight and subsequently, infant mortality.^14^ Successful prevention of these infections reduces the risk of severe maternal anaemia by 38%, low birth weight by 43%, and perinatal mortality by 27% among *pauci – gravidae*.^15^

The World Health Organization (WHO) recommends a package of control and prevention measures to prevent malaria among the most vulnerable group such as pregnant women and children under 5 years of age. Thus, in malaria transmission areas, all pregnant women should sleep under Insecticide-Treated Net (ITN). In addition, in areas of stable transmission of* P. falciparum*, all pregnant women should be given the Intermittent Preventive Treatment (IPT) with *Sulfadoxine–pyrimethamine* (SP) which is one of the key interventions recommended by WHO to bolster the prevention of asymptomatic infections among pregnant women living in moderate to high–risk regions. and has risen since 2010.^16^ Each year, among the approximately 840 million persons at risk of malaria in endemic countries in sub–Saharan Africa, an estimated 35 million pregnant women could benefit from IPT_p_.^17^

World Health Organization has identified potential core elements of monitoring studies of IPT_p_ – SP to include review of ANC (number and timing of IPT_p_ – SP doses) and birth weight data through routine health system records and cross–sectional studies.^14^ Researchers have identified that adherence to these preventive measures in pregnancy helps in reducing the adverse consequences of malaria in pregnancy such as reducing the risk of maternal anaemia, placental parasitaemia and low birth weight.^18^

In 2012, WHO updated its policy to promote initiation of IPT of malaria in pregnancy in all areas with moderate–to–high malaria transmission in Africa, as early as possible in the second trimester.^19^ WHO has observed a slowing of efforts to scale-up intermittent preventive treatment of pregnant women (IPT_p_ Despite the advances in the adoption of the policy by several African countries, the utilisation rate remains low.^17^ World Health Organisation has observed a declining effort to scale up IPT_p_ in a number of African countries. In high – burden countries, IPT_p_ noticeably lags behind other malaria control measures.^20^ As of 2016, thirty six (36) African countries have adopted a policy of providing three or more doses of IPT_p_ with SP to pregnant women.^21^ The Roll Back Malaria partnership initiative set the IPT_p_2 uptake target at 100% by 2015.^22^ However, among the 23 countries that reported in 2016, an estimated 19% of eligible pregnant women received three or more doses of IPT_p_, compared with 18% in 2015 and 13% in 2014.^17^ This does not appear to be due to low levels of ANC attendance. Uncertainty among health workers about IPT_p_ – SP administration for IPT_p_ may have also played a role. Simplified IPT_p_ messages and health worker training have been shown to improve IPT_p_ coverage.^17^

In March of 2015, following several years of extensive support from USAID and UNICEF, Burundi launched IPT_p_ as a National Policy, adding it to the package of services available through the Antenatal Clinic (ANC). In this regard, all pregnant women should receive the first dose of three tablets (IPT_p_1), which providers administer under their direct observation at the ANC facilities from the 15^th^ week of gestation. Recipients of IPT_p_1 should access subsequent doses during each of the scheduled monthly visits to ANC facilities.^14^ Hence all pregnant women should access the second and third doses of IPT_p_ – SP within the 20^th^ and 24^th^ week of pregnancy respectively.^23^

Several researchers have been interested in factors that affect the utilisation of IPT_p_. Lack of knowledge among women and the community about the importance of early ANC booking and IPT_p_ – SP use is one of the challenges that contribute to the low uptake of IPT_p_ in many countries. Other factors include the negative attitude of women toward IPT_p_ – SP, cultural beliefs that inhibit revealing pregnancy early and the distance to the health centre.^4^ Late registration (i.e. within or after pregnancy week 20) and irregular attendance behaviour of pregnant women for ANC services were viewed as a challenge in the efforts to increase IPT_p_ coverage.^25^

In September 2016, 18 months after IPT_p_ implementation, Burundi accounted for 21.0% of pregnant women who completed at least two doses of SP/Fansidar. In 2017, the annual count-down to 2030 report showed that only 13.0% of eligible women in Burundi have received three doses of IPT_p_ – SP during their pregnancy.^26^ In Muramvya province, only 8.1% of eligible pregnant women have completed three doses in 2016, which is below the national level target.^27^

Despite the low rate of IPT_p_ – SP utilisation, ANC attendance is estimated at over 90% nationally in Burundi.^28^ In Muramvya province, 99.3% receive antenatal care from a trained provider while 51.4% perform at least four antenatal visits.^27^ Given these figures, there are indeed challenges in the utilisation and opera-tionalisation of IPT_p_ – SP. In Burundi generally and Muramvya Health District in particular, no study had ever been carried out to assess the level of knowledge of mothers toward IPT_p_–SP and identify determinants of the utilisation of IPT_p_ among pregnant women. Therefore, this study aimed to establish the level of knowledge of pregnant women of Muramvya Health District on IPT_p_ – SP, the utilisation level, and factors affecting its use. This will add to the growing body of knowledge needed for malaria programming for Muramvya Health District and the Ministry of Health by providing strategic information to complement facility-based malaria data sources. The survey will provide community data on key malaria prevention indicators especially the uptake of IPT_p_–SP. The study will help identify individual or client factors that contribute to IPT_p_ uptake. This will provide situational specific evidence that could guide in developing long – lasting interventions. In particular, findings of this study will enable Muramvya Health District to design more effective behaviour change communication campaigns, by designing messages that are tailored to the needs of the people in Muramvya health district.

## METHODS

### Study Area

The study was conducted in Muramvya Health District, 1 of the 2 districts in Muramvya Province, northwest Burundi. Its climate is tropical with 4 seasons, a small rainy season (October to December), a short dry season (January to February), a long rainy season (March to May) and a long dry season (June to September). It covers 42 wards across 2 communes, Bukeye (18) and Muramvya (24). It has 1 general public hospital, 12 Public Primary Health centres and 4 private primary health facilities. The projected population of Muramvya district according to the 2008 census is 186,785 out of which 2.5% represents pregnant women at third term (4,670).^29^

In 2017, consultation due to malaria was 28.8%, acute respiratory infection 23.6%, pneumonia 4.8% and* helminthiasis* 3.7% and approximately 22.3% of pregnant women of Muramvya Health District had at least 1 episode of malaria during their pregnancy in 2017.^30^

**FIGURE 1.**
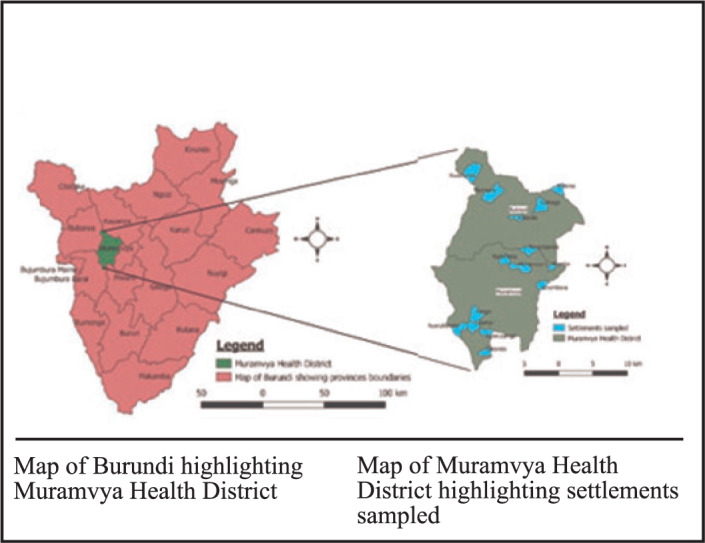
Map Showing Area of Study

### Study Population

The study population included pregnant women who were in their third trimester, living/residing in selected settlements of Muramvya Health District. Pregnant women in their third trimester who were severely ill and or HIV positive on *cotrimoxazole prophylaxis*, and those who refused to grant informed consent were excluded from the study.

### Study Design and Sample Size

Community based cross–sectional survey was conducted in September 2018. The minimum required sample size of this study was determined using single population proportion formula (n=Z2pq/d2) ^31^. Using the utilisation rate of IPT_p_1 (p) of 38.0% ^27^, the confidence interval of 95% (Z=1.96), and the precision (d) of 5% ^32^

$$n=\frac{1.96^{2}*0.38\left(1-0.38\right)}{{\left(0.05\right)}^{2}}=362$$

As the target population is less than 10,000, the minimum sample was adjusted by a finite population correction factor: f=N/ (n+N–1)=4670/(362+4670–1)=.93, then n’=n*f=362*0.93=336. Considering an anticipated no response rate (r) of 10%, n=336+336*10%=370.

### Sampling Procedure

A multi–stage sampling technique was adopted to select 370 pregnant women in their third trimester of pregnancy residing in Muramvya health district.

### First Stage: Selection of District

Muramvya Health province accounts for 2 health districts; Muramvya Health District and Kiganda Health District. The selection of Muramvya Health District was done by simple random sampling using balloting method.

### Second Stage: Selection of Catchment Area

The selected district is divided into 12 catchment areas distributed around 12 public primary health centres and stratified into rural (8) and urban (4). The second stage involved a random selection of 1 urban and 3 rural catchment areas from the 12 catchment areas in Muramvya Health District using simple random sampling technique by balloting. The allocation of the catchment areas was done proportionate to the size of the urban and rural catchment areas.

The names of urban catchment areas were written on pieces of paper, placed in a container and shuffled. 1 catchment area (Muramvya) was randomly selected.

The names of rural catchment areas were also written on pieces of paper, placed in a container and shuffled. Thereafter, 3 catchment areas were randomly selected; hence Ryarusera, Rusarenda and Giko were selected.

### Third Stage: Selection Of Settlements

The list of all the settlements in each of the selected catchment areas was obtained and 25% of settlements in each selected catchment area were selected using simple random sampling technique by balloting. Therefore, a total of 5 settlements were selected from the urban catchment area while a total of 9 settlements were selected from the rural catchment areas.

#### Muramvya Catchment Area:

This catchment area has 19 settlements around Muramvya Health centre. 5 settlements were randomly selected. The names of all 19 settlements were written on pieces of paper, placed in a container and shuffled. After, five settlements were randomly selected; Kadahoka, Rwantamba, Busanga, Muramvya I, Bumba, and Kirembera.

#### Ryarusera catchment area:

This catchment area has 18 settlements around Ryarusera Health centre. 5 settlements were selected. The names of all 18 settlements were written on pieces of paper, placed in a container and shuffled. After, five settlements were randomly selected; Nyarusange, Nyaruhombo, Gasenyi, Rango and Gatando

#### Giko catchment area:

This catchment area has 9 settlements around Giko Health centre. 3 settlements were selected. The names of all 9 settlements were written on pieces of paper, placed in a container and shuffled. After, 3 settlements were randomly selected; Mirama, Gahaga, and Kibande.

#### Rusarenda catchment area:

This catchment area has 8 settlements around Rusarenda Health centre. 2 settlements were selected. The names of all 8 settlements were written on pieces of paper, placed in a container and shuffled. After, 2 settlements were randomly selected; Rusarenda and Muremera.

In total 14 settlements were selected and visited to recruit a total of 370 pregnant women in their third trimester of pregnancy.

### Fourth Stage: Selection of Respondents

The selection of pregnant women in their third trimester of pregnancy to participate in the study in each sampled settlement was done using a systematic sampling technique. Assuming that each pregnant woman in her third trimester of pregnancy represents an eligible household, a list of eligible households was developed in each selected settlement, sampling interval was determined for each settlement and applied accordingly; balloting was employed to determine the first enrolee and the sampling interval was then added to select the subsequent enrolees. When the selected household did not fulfil the inclusion criteria, the immediate neighbourhood was automatically selected. The sample size was distributed among the settlements proportionate to size allocation using the estimate number of pregnant women in their third trimester of pregnancy based on 2008 census

### Data Collection

Information was obtained from respondents using a pre–tested structured questionnaire which was administered to selected consenting pregnant women from each selected settlement who are in their third trimester of pregnancy. The questionnaire was written in English language and translated to Kirundi Language and was administered by 12 trained local interviewers in Kirundi. The questionnaire comprised of questions on socio-demographic characteristics, obstetric history, knowledge of pregnant women to IPT_p_ and its use.

### Data Management

After collection, data was checked for its quality in terms of completeness and errors. Open Data Kit (ODK) Version1.16.0 (ODK-collect, University of Washington, USA) was used for data entry and coding. Data was exported to Microsoft Excel version 2016 (Microsoft Corporation, USA) and cleaned. Univariate analysis was conducted to compute frequencies and proportions. Bivariate analysis at 95% confidence interval was used to compare association between the independent variables and the outcome variables. A p–value equals or less than .05 was considered significant. Multivariate logistic regression analysis was also conducted to determine independent association between some factors that were significantly associated with IPT_p_ utilisation at level of bivariate analysis. Respondents' knowledge on IPT_p_ was also assessed as good, fair, or poor Knowledge. This was determined by asking respondents a set of seven (7) about IPT_p_–SP; – to who it is given, – the purpose of taking it, – when it is recommended, – number of doses pregnant women should take during ANC and interval between doses of IPT_p_–SP. Correct answer was scored 1 while incorrect answer was scored 0 and the maximum obtainable score was 7. The correct responses for each respondent were summed up and the percentage score calculated. Respondents were grouped into 3 groups based on their final score. Those with score equal or above 70% were considered as having good knowledge, those with score between 50% to 69% were considered as having fair knowledge, while those with score less than 50% were considered as having poor knowledge of IPT_p_–SP^14^. Epi–Info version 7.2.2.6 (Epi-Info^TM^, Centers for Disease Control and Prevention (CDC) in Atlanta, Georgia (USA)) was used for data analysis. Tables as well as charts were used to summarise data obtained from the study.

### Ethical Considerations

The ethical approval was obtained from the Burundi National Ethical committee and permission was sought and obtained from Burundi Ministry of Health and Fight against AIDS. The 4 basic ethical principles of justice, beneficence, non-malfeasance, and respect for individual's autonomy were observed. All participants were fully informed about the purpose of the study, potential benefits and the fact that their participation is voluntary. Informed consent was inquired and obtained from all participants before beginning of interview. Furthermore, information sourced was kept confidential. Participants were assured that the information would be used for research purpose only, with access limited to the involved investigators only and stored in a coded password protected computer. Participants were informed that the result of the research will be shared with the District Health Officers, the Ministry of Public Health and Fight against AIDS, the Ahmadu Bello University (ABU) and other stakeholders to support decisions aimed at making the IPT_p_ intervention more responsive.

## RESULTS

A total of 370 pregnant women were interviewed. Their mean age of 28.3±5.6 years and 211 (57.0%) of them were in the age group of 20 to 29 years. Many of the respondents 353 (95.4%) were married. Sixty-nine (18.6%) were nullipara, 252 (68.1%) were multiparous and 49 (13.2%) were grand multiparous. [Table 1].

**TABLE 1.** Socio – Demographic Characteristics of Respondents

Socio – demographic variables	Frequency (N=370)	Percent
**Age group**		
<20	13	3.5
20–29	211	57.0
30–39	137	37.0
>40	9	2.4
**Marital status**		
Married	353	95.4
Single	14	3.8
Widow/Divorced	3	0.8
**Parity**		
0	69	18.7
1–4	252	68.1
≥5	49	13.2
**Education**		
No formal education	95	25.7
Primary	236	63.8
Secondary/Tertiary	39	10.5
**Occupation**		
Farmer	331	89.5
Civil servant	14	3.8
Others	25	6.7
**Location**		
Rural	295	79.7
Urban	75	20.3
**Religion**		
Christianity	363	98.1
Islam	5	1.4
None	2	0.5

### Accessibility to PHC Centres and Time of Registration at Antenatal Clinic

347 respondents (93.8%) attended the clinic and only 67 (18.1%) reside at least 5 km to the clinic. Out of 370 pregnant women interviewed, 343 (92.7%) had visited ANC, at least once during the index pregnancy. More than half of the pregnant women 192 (56.0%) booked during the first trimester. The mean number of ANC visit among the studied subject was 2.2 (±1.0), and only 34 (9.9%) had four (4) visits or more.

### Knowledge of IPT_p_

Among the 370 respondents, 309 (83.5%) were aware of IPT_p_– SP. Among those who were aware of IPT_p_–SP, 289 (93.5%) of them knew that IPT_p_–SP is given to pregnant women at ANC. Majority, 277 (89.6%) of the respondents knew that IPT_p_–SP is given to prevent both mother and baby from contracting malaria. About 179 (57.9%) of the respondents knew that the first dose of IPT_p_–SP should be taken during the second trimester of the pregnancy, and 166 (53.7%) knew the required number of doses of IPT_p_–SP that should be taken during ANC while only 44 (14.2%) knew the monthly interval between doses. Out of 309 who were aware of IPT_p_–SP, majority 252 (81.6%) declared to get information about IPT_p_–SP through ANC staff during ANC visits and 18 (5.8%) through the radio/TV. Only 2 (0.6%) reported that they heard about IPT_p_–SP from Community Healthcare workers. In general, after scoring respondents' knowledge, 98 (31.7%) had good knowledge on IPT_p_–SP. [Table 2]

**TABLE 2.** Knowledge of Pregnant Women of Muramvya Health District on IPT_p_–SP

Knowledge Variable	Frequency (N=309)	Percent
**IPT_p_–SP IPT_p_–SP is normally given to:**		
Pregnant women	289	93.5
HIV positive person	11	3.6
Don't know	9	2.9
**Purpose of giving IPT_p_–SP**		
To prevent mother and baby from malaria	277	89.6
To treat mother and baby from malaria	25	8.1
Don't know	7	2.3
**Time of starting IPT_p_–SP during pregnancy**		
1st trimester	52	16.8
2nd trimester	179	57.9
3rd trimester	21	6.8
Don't know	57	18.4
**Required number of IPT_p_–SP doses in pregnancy**		
One	17	5.5
Two	16	5.2
Three or more	166	53.7
Don't know	110	35.6
**Interval between doses**		
Monthly	45	14.6
Fortnightly	172	55.7
Don't know	92	29.8
**Overall**		
Good	98	31.7
Fair	110	35.6
Poor	101	32.7

### Utilisation of IPT in the Index Pregnancy

**FIGURE 2.**
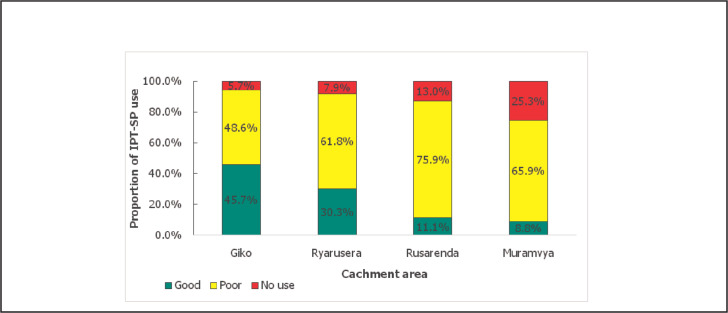
Overall IPT_p_ – SP utilisation by catchment area

Among 370 pregnant women, 310 (83.8%) had taken IPT_p_–SP at least once in the index pregnancy and 301 (97.1%) were among those who have heard of IPT_p_. Among those who were taking IPT_p_, only 76 (24.5%) had completed the minimum required 3 doses; thus, the overall utilisation of IPT_p_–SP among the respondents was good in only 24.5%. According to the description of use by respondents, 310 respondents mentioned that 3 tablets were dispensed to them, and all used the 3 tablets, giving compliance rate of (100.0%). There was difference in IPT_p_– SP utilisation across the studied catchment areas. The Giko catchment area had the highest proportion (45.7%) of pregnant women who had taken at least 3 doses of IPT while Muramvya has the lowest proportion (8.8%) [Figure 2].

### Factors Affecting the IPT_p_–SP Uptake

Having any formal education was found to be positively associated with the uptake of IPT_p_–SP by the pregnant women. Living at less than 5km from the health facility (OR=2.1, 95% CI [1.1–2.2], P=.039), was positively associated with the uptake of IPT_p_–SP by the pregnant women, and having good knowledge on IPT_p_–SP (OR=57.0, 95% CI [16.1–353.1], P<.001), were found to be positively associated with the uptake of IPT_p_–SP by the pregnant women.

To assess independents determinants influencing the uptake of IPT_p_–SP, determinants with a P≤ .2 in bivariate analysis were put into unconditional logistic regression. Education, parity, having good knowledge on IPT_p_–SP, and residing less than 5km from the Health Facility were found to be independent determinants of IPT_p_–SP uptake. [Table 3 **and** 4]

**TABLE 3.** Factors Affecting the IPT_p_ – SP Uptake in Muramvya Health District

Variable	IPT_p_ uptake		OR [95%CI]	P– value
	Yes Freq (%)	No Freq (%)		
**Age (years)**				
<35	243 (84.4)	45 (15.6)	1.2 [0.6 – 2.3]	.683
≥35	67 (81.7)	15 (18.3)		
**Marital status**				
Married	297 (84.1)	56 (15.9)	1.6 [0.4 – 5.0]	.617
Not married	13 (76.5)	4 (23.5)		
**Parity**				
0–4	231 (86.2)	37 (13.8)	1.8 [1.0 – 3.2]	.059
≥5	79 (77.5)	23 (22.5)		
**Location**				
Urban	68 (90.7)	7 (9.3)	2.1 [1.0 – 5.2]	.102
Rural	242 (82.0)	53 (18.0)		
**Education**				
Formal	238 (86.5)	37 (13.5)	2.1 [1.1 – 3.7]	.022
Non – formal	72 (75.8)	23 (24.2)		
**Occupation**				
Farmer	271 (81.9)	60 (18.1)	0.0 [0.0 – 0.4]	.007
Others	39 (100.0)	0 (0.0)		
**Distance from home to ANC**				
<5km	260 (85.8)	43 (14.2)	2.1 [1.1 – 2.2]	.039
≤5km	50 (74.6)	17 (25.4)		
**Mode of transportation**				
Foot	287 (82.7)	60 (17.3)	0.0 [0.0 – 0.6]	.060
Vehicle/bike	23 (100.0)	0 (0.0)		
**Knowledge on IPT_p_–SP**				
Good	97 (99.0)	1 (1.0)	57.0 [16.1 – 353.1]	<.001
Poor	104 (64.2)	58 (35.8)		

**TABLE 4.** Unconditional Logistic Regression for Independent Determinants of the Uptake of IPT_p_–SP

Variables	aOR	95%CI	P–value
Education (Formal/No–formal)	2.5	1.2–5.2	.016
Parity (0-4/≥5)	2.1	1.1–4.2	.033
Knowledge on IPT_p_ – SP (Good/Poor)	68.3	15.5–300.2	<.001
Location (Urban/Rural)	1.1	0.4–2.9	.834
Distance from home to ANC (≤5km/>5km)	4.1	1.7–9.6	.001
Occupation (Farmer/Other)	2.0	0.7–6.0	.208

### Factors Affecting the Good Utilisation of IPT_p_ – SP

Gestational age at first ANC (OR=2.8, 95% CI [1.3–6.7], p– value=.011), living in rural area (OR=2.2, 95% CI [1.1–4.7], P=.048), and having formal education were found to be determinants of good IPT_p_–SP utilisation. Again, having good knowledge on IPT_p_ (OR=3.4, 95% CI = [1.8–7.0], P<.001), was found to be a determinant of good utilisation of IPT_p_–SP. After controlling for possible confounders, Education (aOR=5.0, 95% CI [2.1–24.3], P<.001), gestational age at first ANC visit (aOR=3.3, 95% CI [1.4–7.7], P=.005), and knowledge on IPT_p_ (aOR=2.5, 95% CI [1.4–4.5], P=.010) were found to be independent determinants of good utilisation of IPT_p_–SP. [Table 5]

**TABLE 5.** Association Between Respondents' Socio–Demographic Characteristics, Other Factors and Good Utilisation of IPT_p_–SP

Variable	IPT_p_ utilisation		OR [95%CI]	P-value	aOR [95%CI]	P-value
	Good Poor Freq (%)	Freq (%)				
**Age (years)**						
<35	62 (25.5)	181 (74.5)	1.3 [0.7 – 2.5]	.437	-	
≥35	14 (20.9)	53 (79.1)				
**Gestational age at 1stANC visit**						
Early	68 (28.0)	175 (72.0)	2.8 [1.3 – 6.7]	.011	3.3 [1.4 -7.7]	.005
Late	8 (11.9)	59 (88.1)				
**Marital status**						
Married	74 (24.9)	223 (75.1)	1.8 [0.4 – 8.4]	.651	-	
Not married	2 (15.4)	11 (84.6)				
**Parity**						
0–4	55 (23.8)	176 (76.2)	0.9 [0.5 – 1.6]	.731	-	
≥5	21 (26.6)	58 (73.4)				
**Location**						
Rural	66 (27.3)	176 (72.7)	2.2 [1.1 – 4.7]	.048	1.7 [0.8–3.9]	.184
Urban	10 (14.7)	58 (85.3)				
**Education**						
Formal	69 (29.0)	169 (71.0)	3.7 [1.7 – 9.3]	.001	5.0 [2.1–12.3]	<.001
Non–formal	7 (9.7)	65 (90.3)				
**Distance from home to ANC**						
≤ 5 km	64 (24.6)	196 (75.4)	1.03 [0.5 – 2.2]	.999	-	
>5 km	12 (24.0)	38 (76.0)				
**Knowledge on IPT**						
Good	64 (31.1)	142 (68.9)	3.4 [1.8 – 7.0]	<.001	2.5 [1.4 – 4.5]	.002
Poor	12 (11.5)	92 (88.5)				

## DISCUSSION

Our findings show that most of the pregnant women interviewed were aware of IPT_p_–SP and knew that IPT_p_–SP is given to pregnant women at ANC to prevent mother and baby from malaria. However, the overall knowledge of IPT was good only in 31.7% of pregnant women who were aware of it. Studies from Nigeria, Ghana ^14,34–36^ have also shown the low level of knowledge of IPT–SP among pregnant women. However, about 70% of respondents had good knowledge on IPT_p_ in Ghana in 2016. This difference in findings may be explained by the time when the study was conducted. Our study was conducted in 2018, only three years of implementation of IPT_p_ as part of ANC package while the study of Hajira *et al* was conducted in 2015, after eight years of IPT_p_ implementation in Ghana.^37^ Most of the respondents did not know the number of required doses and the regular interval at which IPT_p_–SP should be taken during ANC which is the same with the result of the study conducted in Hohoe Municipality of Ghana where they found that majority of the pregnant women did not know the correct interval.^36^ Some of these disparities noted from this study could be explained by variations in literacy levels, place of residence, methodology, or timing of the studies.

This knowledge gap may also be due to lack of personal and institutional updates on new interventions in preventing malaria during pregnancy in the district or even from the gap in number of staffs who might not have enough time to explain to all the pregnant women during ANC visit. Thus, the key informants interviewed complained that the staff is not enough, this may have influenced the time allocated to health talks. It may also be as a result of lack of budgetary support in training of Community Healthcare Workers (CHCWs) who could sensitise communities on IPT_p_–SP, and others malaria control strategies. It was also found that the source of information was mostly from healthcare workers during ANC. This may explain the low implication of CHCWs in sensitisation of community on malaria and its preventive measures especially the IPT_p_–SP. The evidence from this study indicate that 83.8% of women have taken at least 1 dose of IPT_p_–SP during the index pregnancy. Our results differ from 2016 to 2017 Burundi Demographic and Health Survey (BDHS) which reported that proportion of women receiving at least 1 dose SP during their third trimester of their pregnancy was 18,4% in Muramvya province lodging Muramvya Health District.^27^ This may be explained by the time difference that there could have been improvement in accessing and utilising health care service through time. 2016 to 2017 BDHS was conducted after 18 months of IPT_p_–SP implementation while our study was conducted 40 months after. The IPT_p_– SP uptake rate in our study is also higher than the findings of the study conducted in rural areas of the western part of Kenya in 2005, where only 41% had taken at least 1 dose of SP in their third trimester of pregnancy^14^, and the study conducted in 2014 to 2015, where 68.0% of pregnant women were taking IPT–SP during their pregnancy in Tanzania.^38^ The study conducted in Cote d'Ivoire showed that 83.7% women received ≥1 dose of IPT–SP as prophylaxis against malaria during their pregnancy.^39^ However, the SP uptake in this study is lower than that of Ghana, which revealed that 98.5% of the pregnant women of Sunyani Municipality of Ghana received at least one (1) dose of SP during the current pregnancy.^37^ These differences may have been mainly due to the time between the implementation of IPT_p_–SP programme and the conduction of this study World Health Organization has also revealed the increase in the IPT_p_–SP uptake through years; the proportion pregnant women who took at least 1 dose of SP has raised from 45% in 2010 to 56% in 2016^40^ and dropped down to 54% in 2017.^4^ The gestational age at the first ANC visit is believed to be very important to the coverage of IPT_p_–SP. Early registration increases one's opportunity of receiving the recommended doses of SP, given ANC is attended regularly and SP is available. Late first ANC attendance has been found to be a factor of incomplete doses of IPT_p_–SP.^14^ In this study, the median gestational age of first ANC visit was found to be 11 weeks (range 3 to 31), majority of the respondents booked during the first and second trimesters (50.6% and 40.8% respectively). Our findings are similar to a study conducted in Arusha Region of Tanzania where 39.4% of the pregnant women registered their first ANC attendance in the first trimester while 60.6% registered in or after their second trimester.^41^ However, the findings of this study differ from those of Mutengene Health Area, Mt Cameroon where 2.2%, 59.7%, and 38.1% enrolled in the first, second and third trimester respectively.^25^ Our results also are different from the findings from Kano, North west Nigeria where the majority of women booked in the second trimester (13 to 24 weeks).^35^ This means that pregnant women in Muramvya Health District start attending the ANC early enough which could allow them to receive the recommended 3 doses of SP according to National Strategy. This early ANC booking may be explained by the Burundi government strategy of free healthcare services to pregnant women. In addition, the district provides a kind of motivation for pregnant women who book early for their first ANC visits as declared by our key informants. However, despite the early first ANC visit, the mean number of ANC visits among pregnant women interviewed was 2.1±1.1 (range 1 to 6). Most of the women 41.1% had two (2) visits, those who had 3 visits were 27.7% and only 9.9% had four (4) visits or more. On this, the findings are similar with those from Jigawa where the mean number of ANC visits was found to be 2.7 (range 1 to 6). Most of the women 51.2% having two (2) visits and only 9.3% had four (4) visits.^14^ In a study on Implementation of intermittent preventive treatment with *sulphadoxine-pyrimethamine* for control of malaria in pregnancy conducted in Kisumu, western Kenya, where about 50% of the women attended their first ANC in the third trimester, about 25% received 2 doses of SP.^42^ The results of this study showed that, among those women who have taken at least 1 dose SP, only 24.5% have completed the 3 recommended doses which is considered as good utilisation of IPT_p_–SP. This rate is higher than the report of 2016 to 2017 BDHS where only 8.1% of pregnant women of Muramvya province reported having completed the 3 doses of IPT^27^, and Countdown to 2030 report where about 13.0% of pregnant women received 3 doses or more in 2016 in Burundi .^26^ This may have been due to the improvement overtime from the beginning of the programme. Our findings are close to the WHO report on malaria where 22% of eligible pregnant women received at least 3 doses of IPT_p_–SP in 2017.^4^ The results of this study showed differences between others studies conducted in others countries. In 2016, the study conducted in Jigawa state of Nigeria found that only 1.4% pregnant women have received at least 2 doses of SP.^14^ This finding is in contrast from a study in Ghana which showed that not less than 70% received at least 3 doses of SP^37^, and the one conducted in Arusha Region, Tanzania where 48.4% had received >3 doses of SP-IPT_p_^41^. These differences may be due to time differences. The poor utilisation of IPT_p_–SP among pregnant women in this study suggests that many pregnant women are not benefiting from the laudable initiative aimed at reducing the level of maternal and neonatal mortality associated with malaria in pregnancy, despite the fact that majority of these women register early for antenatal care. Bearing in mind that Burundi rank high among countries with high maternal and neonatal mortality rate.^43^

Different determinants have been identified to be independently associated with utilisation/non utilisation and the good/poor utilisation of IPT_p_–SP. These are; - education level, - parity, -distance from home to the health centre, - gestational age at the first ANC visit, and the level of knowledge on IPT_p_–SP. Education improves health, while health improves learning potential. Education and health complement, enhance and support each other; together, they serve as the foundation for a better world.^44^ Increasing women's education increases antenatal healthcare use, potentially owing to changes in women's cognitive skills, economic resources, and autonomy.^45^ use, potentially owing to changes in women's cognitive skills, economic resources, and autonomy.^45^ In Rubavu district of Rwanda, education was associated with the utilisation of health service, in 2012.^46^ This study revealed that having any formal education was positively associated with the utilisation and the completion of at least 3 doses of SP. This finding agrees with the study conducted in Tanzania in 2012^47^ and 2017^41^, where education associated with uptake of >3 doses of. From 1990 to 2013, education was one of key determinants of IPT_p_ coverage.^48^ However, the study conducted in Sagamu, rural town in Western Nigeria, found education level to be not associated with the utilisation of IPT_p_–SP. This difference may have been to the study population, in that study, about 92.2% have formal education^49^ contrarily to our study. Even thought it was not statistically significant, the study conducted in Pobè-Adja-Ouèrè-Kétou health zone in Benin show the poor utilisation of IPT_p_– SP among pregnant women with no any formal education.^50^

In this study, it was found that parity was associated with IPT utilisation. Therefore, pregnant women who have taken at least 1 dose of SP were more likely to have low parity than those who did not. However, among those who were using IPT_p_–SP, the parity was not associated with the completion of the minimum of 3 doses of SP. The study conducted by in Jigawa State, Nigeria and in Pobè-Adja-Ouèrè-Kétou health zone in Benin found that parity were not associated with the coverage of IPT_p_–SP.^14, 50^ However, in South-West Nigeria, the study conducted in 2012 reported the increasing uptake of IPT_p_ with increasing parity.^49^

The distance from home to the health centre may play a role in IPT_p_–SP coverage.^51^ This was confirmed in our study where pregnant women who have taken at least 1 dose of IPT_p_–SP were more likely to be living less than 5km from the health centre.. However, it was not affecting the completion of minimum 3 doses among those who were utilising the IPT_p_–SP. The study in Jigawa state of Nigeria found no relationship between distance and uptake of IPT_p_–SP.^14^ This difference may have been to the study population because, they used the ANC based while our methodology was community based. In the study conducted in Mali in 2016, health workers considered non-attendance or late attendance at ANC among the top barriers to IPT_p_–SP up-take while women and family members saw ANC as valuable, they noted cost and distance as significant barriers to healthcare seeking.^52^ The study conducted in Benin in 2012 did not found any association between the distance and the IPT_p_–SP coverage.^50^

Despite the median gestational age at first ANC of 11 weeks (range 3 to 31) and the high proportion of women who took the first dose, which is an opportunity for the pregnant women to receive the recommended 3 or more doses of IPT_p_–SP, the utilisation of SP still low. However, early first ANC attendance was found to positively affect the completion of the minimum 3 doses of IPT_p_–SP. Thus, all key informants stated that the many challenges that affect the rate of utilisation of IPT_p_–SP were the late first ANC booking and the irregularity in subsequent ANC visits. This finding highlights the need for appropriate dissemination of the current intermittent preventive treatment for malaria guidelines and further scrutiny of the quality of the antenatal care services provided at the primary health care centres by healthcare workers and particularly in the communities by CHCWs. The analysis from 5 Africa countries have shown that the facilities having IPT_p_ guidelines and having implemented IPT_p_ as part of their routine ANC services was major determinant of IPT_p_ delivery.^53^ This is to ensure that opportunities for malaria prevention are not missed because of the weaknesses of the health care system. This position is particularly important because ANC attendance rates even in rural communities in Burundi are usually high.^27,28^ Early ANC booking was also found to be positively associated with the utilisation of IPT_p_–SP in Bukoba, Tanzania^54^ and in Ekiti State of Nigeria.^34^ The study conducted in 2016 also confirmed that pregnant women of the Pobè-Adja-Ouèrè-Kétou health zone in Benin who attended the ANC at 3 months or less were more likely to have been given at least 1 dose IPT_p_–SP.^50^ In Ghana, in 2015, the study established the relationship between gestational age at first ANC attendance and the number of doses given.^55^ In other hand, the study conducted in Tanzania in 2015 didn't find the significant relationship between ANC start date and doses of SP received.^56^

The level of knowledge on IPT_p_–SP was found to be an independent determinant of the completion of required doses. This is similar to the findings of the study conducted in a rural town in Western Nigeria, and in Bugiri District in south-eastern Uganda.^49, 57,58^ Others studies conducted in Tanzania in 2015,^56^ have not established the link between the knowledge on IPT_p_–SP and its utilisation. Other individuals' factors like age, marital status, to be resident in urban or rural area were not found to affect the utilisation or the completion of minimum 3 doses of SP. The free maternal health care policy being implemented in the district could have helped to overcome these factors as barriers to accessing healthcare through the ANCs. This was in agreement with previous study in Tanzania ^54^ and Jigawa state of Nigeria^14^ where individual or client factors were not found to be associated with second dose SP administration. A study done in Malawi also showed that age was not associated the utilisation of IPT_p_– SP.^59^ In Kano and Ekiti States, Nigeria, studies conducted didn't find association between IPT_p_–SP uptake and respondent age which is similar to the finding from this study.^34,35^

## CONCLUSION

This study revealed a high level of awareness, however, the level of knowledge about IPT_p_–SP was low. The findings of this study show the high rate of women receiving the first dose of SP; however, the rate of women receiving the minimum 3 doses was very low. The prevalent factors that could affect the utilisation of IPT_p_–SP in Muramvya Health District were educational level, parity, distance from home to the health centre, gestational age at the first ANC visit, and the level of knowledge on IPT_p_–SP. The high antenatal attendance of pregnant women and the availability of the drug are important opportunities available for improvement of IPT_p_–SP program in order to achieve good utilisation of IPT_p_-SP that can help in reducing malaria in pregnancy generally in Burundi and Muramvya Health District in particular.

### Limitations

The respondents may not have recalled all that happened during their ANC visits leading to recall bias. However, this was minimized by showing samples of SP to them in order for them to relate their responses to the drug in question.
